# Estimated Savings After Stopping Tyrosine Kinase Inhibitor Treatment Among Patients With Chronic Myeloid Leukemia

**DOI:** 10.1001/jamanetworkopen.2023.47950

**Published:** 2023-12-18

**Authors:** Aaron N. Winn, Ehab Atallah, Jorge Cortes, Michael W. N. Deininger, Vamsi Kota, Richard A. Larson, Joseph O. Moore, Michael J. Mauro, Vivian G. Oehler, Javier Pinilla-Ibarz, Jerald P. Radich, Neil P. Shah, James E. Thompson, Kathryn E. Flynn

**Affiliations:** 1 Department of Health Systems, Outcomes and Policy, School of Pharmacy, University of Illinois at Chicago; 2 Department of Medicine, Medical College of Wisconsin, Milwaukee; 3 Georgia Cancer Center, Augusta University Medical Center, Augusta; 4 Department of Medicine and Comprehensive Cancer Center, University of Chicago, Chicago, Illinois; 5 Duke University School of Medicine, Durham, North Carolina; 6 Memorial Sloan Kettering Cancer Center, New York, New York; 7 Fred Hutchinson Cancer Research Center, Seattle, Washington; 8 H. Lee Moffitt Cancer Center & Research Institute, Tampa, Florida; 9 Department of Medicine, University of California at San Francisco; 10 Roswell Park Comprehensive Cancer Center, Buffalo, New York

## Abstract

**Importance:**

Patients with chronic myeloid leukemia (CML) who have a sustained deep molecular response using tyrosine kinase inhibitors (TKIs) can safely attempt to stop their use. As these medications are very costly, this change in treatment protocols may result in large savings.

**Objective:**

To estimate future savings from attempting to stop TKI use among patients with CML who have deep molecular response.

**Design, Setting, and Participants:**

A microsimulation model was developed for this decision analytical modeling study to estimate costs for US adults moving from using a TKI, to attempting discontinuation and then reinitiating TKI therapy, if clinically appropriate. Estimates were calculated for US patients who currently have CML and simulated newly diagnosed cohorts of patients over the next 30 years.

**Exposure:**

Attempting to stop using a TKI.

**Main Outcomes and Measures:**

Estimated savings after attempted discontinuation of TKI use.

**Results:**

A simulated population of individuals with CML in 2018 and future populations were created using estimates from the SEER*Explorer website. The median age at diagnosis was 66 years for men and 65 years for women. Between 2022 and 2052, the savings associated with eligible patients attempting discontinuation of TKI therapy was estimated at more than $30 billion among those currently diagnosed and over $15 billion among those who will develop CML in the future, for a total savings of over $54 billion by 2052 for drug treatment and polymerase chain reaction testing. The estimate is conservative as it does not account for complications and other health care–associated costs for patients continuing TKI therapy.

**Conclusions and Relevance:**

The findings of this decision analytical modeling study of patients with CML suggest that attempting discontinuation of TKI therapy could save over $54 billion during the next 30 years. Further education for patients and physicians is needed to safely increase the number of patients who can successfully attain treatment-free remission.

## Key Points

**Question:**

What is the estimated population-level financial effect of discontinuing tyrosine kinase inhibitor (TKI) treatment in patients with chronic myeloid leukemia (CML) who have a sustained deep molecular response?

**Findings:**

In this decision analytical modeling study of the current US population with CML, a microsimulation model was used to estimate future health care spending. The findings indicate that an estimated $50 billion could be saved by eligible patients who attempt to stop TKI therapy.

**Meaning:**

These findings suggest that attempting to stop TKI therapy could improve quality of life for patients with CML and may result in a large reduction in health care costs.

## Teaser

This decision analytical modeling study estimates the financial outcome of discontinuation of tyrosine kinase inhibitor (TKI) therapy among US adult patients with chronic myeloid leukemia (CML) who experience deep molecular response to TKIs.

## Introduction

Since the introduction of tyrosine kinase inhibitors (TKIs), care for patients with chronic myeloid leukemia (CML) has radically changed, and survival of patients with CML is now similar to survival in the general population without CML.^1^ However, TKIs remain very expensive, resulting in a financial burden on patients and the health care system.^2^ For patients with a sustained deep molecular response (MR4 or better; BCR::ABL1 ≤ 0.01% on the International Scale), attempting discontinuation of TKI therapy is safe. Discontinuation is associated with sustained treatment-free remission (TFR) for about 50% of patients who attempt it.^3,4,5^ The Life After Stopping TKIs (LAST) study^6^ was the first prospective US-only trial to evaluate TFR; at 3 years, 60.8% sustained discontinuation of TKI therapy. Attempting to discontinue TKI therapy requires additional laboratory tests to monitor molecular response. Herein, we used findings from the LAST study to estimate spending on drugs and testing associated with trying to discontinue TKI therapy among all eligible adult patients in the US. We constructed a model of CML drug and polymerase chain reaction (PCR) costs that account for savings from reduced TKI use during TFR, increased testing during TFR, and increased costs associated with reinitiating TKI treatment after a failed discontinuation attempt. Herein, we estimate the changes in health care spending after attempting to discontinue TKI therapy among eligible US adults over the next 30 years.

## Methods

### Population

This decision analytical modeling study was approved by the Medical College of Wisconsin Institutional Review Board. We used the Consolidated Health Economic Evaluation Reporting Standards (CHEERS) checklist to guide the development of this model. To estimate the future drug and PCR spending for patients with CML in the US, we accounted for the current population with CML (prevalent cohort) and estimated new cases of CML (incident cohort) expected over time. We created the prevalent cohort based on the population of individuals with CML in 2018 using estimates from the SEER*Explorer interactive website.^7^ We created the incident cohort by taking the current US population and annually diagnosing individuals with CML based on age and sex incidence rates.^7^ For those who were not diagnosed, we allowed individuals to die based on age- and sex-specific US mortality tables.^8^ We aged those not diagnosed with CML and who did not die each year and repeated the process, creating an incidence cohort each year.

Consistent with current clinical practice guidelines,^9^ we required patients to have used TKIs for at least 3 years and achieved MR4 for at least 2 years to be eligible to attempt TKI discontinuation. For the prevalent cohort, we estimated that 30% of patients using a first-generation TKI would be at MR4, and 50% of patients using a second-generation TKI would be at MR4.^10^ For the incident cohort, we estimated the time to reach MR4 within 5 years after diagnosis by fitting a parametric time-to-event model for those using a first- or second-generation TKI to match previously published trial-based time-to-MR4 curves.^11^ If patients were not eligible at year 5, we made the conservative assumption that they would not become eligible; however, some studies find that some patients will reach MR4 after 5 years.^12^

### Simulation Model

With a 1-month cycle length, we developed a microsimulation model of patients with sustained deep molecular response to compare drug and PCR costs of continuing and attempting to stop TKI therapy. Based on the LAST study and other trials, we assumed that attempting discontinuation with increased monitoring does not result in disease progression or changes in mortality rates. Costs of health care services used were assigned based on current TKI use and associated expected testing. Consistent with other forecasting studies of absolute projections, we did not report SEs or 95% CIs.^13,14,15^

### Model Components

#### Use of TKIs

Based on a commercial claims (MarketScan) observational database study,^16^ we assumed that 54% of patients start therapy with a second-generation TKI. We estimated the time to TKI therapy discontinuation for patients who attempt discontinuation using parametric time-to-event models. Using data from the LAST study, we estimated time-to-event models from discontinuation to reinitiation of treatment. Given the different hazard functions in the early period (within 18 months of discontinuation) and the late period (after 18 months of discontinuation), we fit 2 models. We fit a parametric time-to-event model (exponential, Weibull, and Gompertz) for both periods and selected the model with the best Akaike and Bayesian information criteria. In the early period, we used a Gompertz model, whose hazard can take on different shapes to ensure it matches the underlying data; for the late period, we used the exponential model, which has a constant hazard (eFigures 1 and 2 in Supplement 1 provide a comparison between models and empirical survival estimates that show curves that are very similar to the empirical data). For patients who do not attempt TKI discontinuation because they do not reach MR4, we assumed they were taking a TKI.

#### Mortality

We used US life tables to estimate a patient’s risk of death. We assumed that patients who reach MR4 survive similarly to the general population.^1,17^

#### Costs

After TKI therapy discontinuation, patients are tested for CML recurrence monthly for the first 6 months, bimonthly for the next 18 months, and then every 3 months for the entirety of their lives. For the comparison group, those who never attempted to discontinue TKI therapy are tested every 3 months. We estimated spending associated with first- and second-generation TKIs based on previously published findings using MarketScan (Table).^18^ We assumed that the cost of a PCR test was US $144 based on the Medicare laboratory fee schedule.^19^

**Table.** Model Parameters

Parameter	Value	Source
Cost of first-generation TKI	$1375	Nguyen et al,^18^ 2020
Cost of second-generation TKI	$12 530	Nguyen et al,^18^ 2020
Cost of PCR test	$144	Centers for Medicare & Medicaid Services,^19^ 2022
Initiation of a second-generation TKI, %	54	Cole et al,^16^ 2020
MR4 among the prevalence cohort using first-generation TKIs, %	30	Atallah and Schiffer,^10^ 2020
MR4 among the prevalence cohort using second-generation TKIs, %	50	Atallah and Schiffer,^10^ 2020
Time to MR4 among newly diagnosed patients using a first-generation TKI		
Gompertz model γ	0.0202007	Hochhaus et al,^11^ 2016
Gompertz model λ	−5.801135	
Time to MR4 among newly diagnosed patients using a second-generation TKI, exponential model, l	−4.43556	Hochhaus et al,^11^ 2016
Time to TKI restart		
2-18 mo		Atallah et al,^6^ 2021^a^
Gompertz model γ	0.05297216	
Gompertz model λ	−0.11756448	
18-60 mo, exponential model, l	−5.9341885	

Abbreviations: MR4, deep molecular response; PCR, polymerase chain reaction; TKI, tyrosine kinase inhibitor.

^a^ Estimated from the Life After Stopping TKIs (LAST) study.

### Sensitivity Analysis

Prior research has found that the US population with CML has a risk of death higher than that of the general population.^20^ While this is not true among those that have reached MR4, as an upper bound, we estimated how doubling the risk of mortality might affect the savings.

## Results

**Figure 1.**
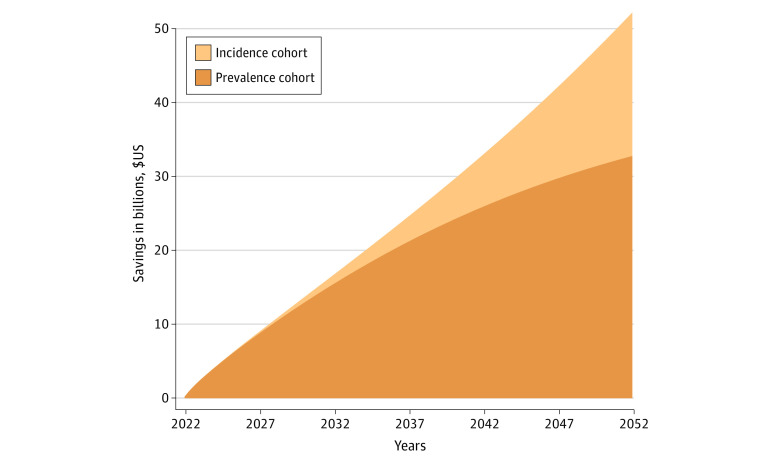
Estimated Savings Associated With Attempting to Stop Tyrosine Kinase Inhibitor Use Among Patients With Chronic Myeloid Leukemia in Deep Molecular Response

**Figure 2.**
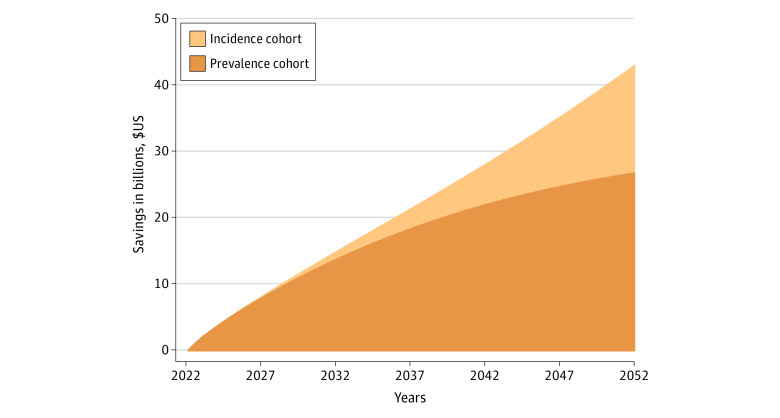
Sensitivity Analysis of Estimated Savings Associated With Attempting to Stop Tyrosine Kinase Inhibitor Use Among Patients With Chronic Myeloid Leukemia in Deep Molecular Response The analysis assumed that the mortality rate was twice the national average in the US.

The median age at diagnosis was 66 years for men and 65 years for women. We found that the cumulative savings for attempting to discontinue TKI therapy for eligible patients was over $15 billion within 10 years (Figure 1). These savings continued to grow; at 30 years, the estimated savings were over $54 billion. Sensitivity analyses assuming patients’ mortality rate was double the national average found savings at $43 billion (Figure 2).

## Discussion

In this decision analytical modeling study, we estimate that attempting to discontinue TKI therapy for patients with CML who achieved MR4 could result in considerable savings to the health care system. Taken with other research, which has found that patients’ quality of life improves when they discontinue TKI therapy,^6,21^ that patients define a cure as discontinued TKI therapy,^22^ and that a patient’s disease does not progress even if they have to reinitiate treatment,^3,4,5,6^ it is clear that widespread TKI therapy discontinuation is beneficial for eligible patients.

### Limitations 

This study has some limitations. First, we made the conservative assumption that CML incidence will remain constant over time. However, other investigators^23^ have documented a 1.1% increase in incidence rates over time. Second, we assumed that the proportion of patients starting second-generation TKI therapy would remain constant over time. However, if more patients use second-generation TKIs over time, our results are conservative since second-generation TKIs are more likely to result in patients achieving MR4 (increasing the size of the eligible population) and are more expensive (increasing the cost per patient). Third, first-generation TKIs have become and second-generation TKIs will soon become generic, changing the cost-savings estimates. Fourth, patients may not require monitoring as frequently, which would lower the costs of attempting to stop treatment.

## Conclusions 

Given the potential savings and improvements in health from attempting to stop use of TKIs as suggested by this decision analytical modeling study, future research should focus on the most effective treatment approaches that allow patients to discontinue TKI therapy. Further education for patients and physicians is needed to safely increase the number of patients who can successfully attain TFR.^24^

## Notes

Supplement 1. **eFigure 1.** Time to TKI Discontinuation Comparisons Between Nonparametric and Parametric Model in the First 18 Months Based on the LAST Study **eFigure 2.** Time to TKI Discontinuation Comparison Between Nonparametric and Parametric Model After Month 18 Based on the LAST Study

Supplement 2.
Data Sharing Statement
