# Supplementary material for: Estimated Savings After Stopping Tyrosine Kinase Inhibitor Treatment Among Patients With Chronic Myeloid Leukemia
Source: JAMA Netw Open. 2023 Dec 18;6(12):e2347950. doi: 10.1001/jamanetworkopen.2023.47950 (PMC10728762; doi:10.1001/jamanetworkopen.2023.47950)
Supplement: Supplement 1. — eFigure 1. Time to TKI Discontinuation Comparisons Between Nonparametric and Parametric Model in the First 18 Months Based on the LAST Study eFigure 2. Time to TKI Discontinuation Comparison Between Nonparametric and Parametric Model After Month 18 Based on the LAST Study [file jamanetwopen-e2347950-s001.pdf]

Supplementary Online Content

Winn AN, Atallah E, Cortes J, et al. Estimated savings after stopping Tyrosine Kinase Inhibitor treatment among patients with chronic myeloid leukemia. *JAMA Netw Open*. 2023;6(12):e2347950. doi: 0.1001/jamanetworkopen.2023.47950

eFigure 1. Time to TKI Discontinuation Comparisons Between Nonparametric and Parametric Model in the First 18 Months Based on the LAST Study

eFigure 2. Time to TKI Discontinuation Comparison Between Nonparametric and Parametric Model After Month 18 Based on the LAST Stu

This supplementary material has been provided by the authors to give readers additional information about their work.

© 2023 Winn AN et al. *JAMA Network Open*.

eFigure 1. Time to TKI Discontinuation Comparisons Between Nonparametric and Parametric Model in the First 18 Months Based on the LAST Study

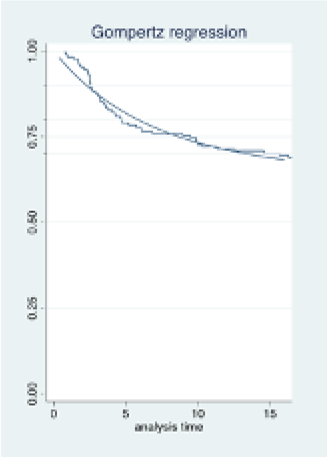

eFigure 2. Time to TKI Discontinuation Comparison Between Nonparametric and Parametric Model After Month 18 Based on the LAST Study

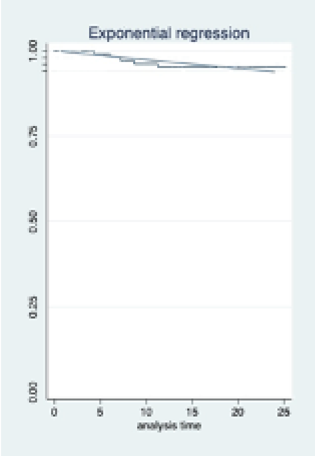

© 2023 Winn AN et al. *JAMA Network Open*.
