# Supplementary material for: Estimated Savings After Stopping Tyrosine Kinase Inhibitor Treatment Among Patients With Chronic Myeloid Leukemia
Source: JAMA Netw Open. 2023 Dec 18;6(12):e2347950. doi: 10.1001/jamanetworkopen.2023.47950 (PMC10728762; doi:10.1001/jamanetworkopen.2023.47950)
Supplement: Supplement 2. — Data Sharing Statement [file jamanetwopen-e2347950-s002.pdf]

Data Sharing Statement

Winn. Estimated Savings After Stopping Tyrosine Kinase Inhibitor Treatment Among Patients With Chronic Myeloid Leukemia. *JAMA Netw Open*. Published December 18, 2023. doi:10.1001/jamanetworkopen.2023.47950

Data
Data available: No

Additional Information
Explanation for why data not available: Decision model code will be freely available if requested and reviewed by the authors.
